# Graphene Oxide Oxygen Content Affects Physical and Biological Properties of Scaffolds Based on Chitosan/Graphene Oxide Conjugates

**DOI:** 10.3390/ma12071142

**Published:** 2019-04-08

**Authors:** Iolanda Francolini, Elena Perugini, Ilaria Silvestro, Mariangela Lopreiato, Anna Scotto d’Abusco, Federica Valentini, Ernesto Placidi, Fabrizio Arciprete, Andrea Martinelli, Antonella Piozzi

**Affiliations:** 1 Department of Chemistry, Sapienza University of Rome, P.le A. Moro, 5, 00185 Rome, Italy; iolanda.francolini@uniroma1.it (I.F.); peruginielena80@gmail.com (E.P.); ilaria.silvestro@uniroma1.it (I.S.); andrea.martinelli@uniroma1.it (A.M.); 2 Department of Biochemical Sciences, Sapienza University of Rome, P.le A. Moro, 5, 00185 Rome, Italy; mariangela.lopreiato@uniroma1.it (M.L.); anna.scottodabusco@uniroma1.it (A.S.d.); 3 Department of Chemical Science and Technologies, University of Rome Tor Vergata, Via della Ricerca Scientifica, 00133 Rome, Italy; federica.valentini@uniroma2.it; 4 Department of Physics, University of Rome Tor Vergata, Via della Ricerca Scientifica, 00133 Rome, Italy; ernesto.placidi@roma2.infn.it (E.P.); fabrizio.arciprete@roma2.infn.it (F.A.); 5 CNR-ISM, Via Fosso del Cavaliere 100, I-00133 Rome, Italy

**Keywords:** graphene oxide, chitosan, composites, scaffolds, tissue engineering

## Abstract

Tissue engineering is a highly interdisciplinary field of medicine aiming at regenerating damaged tissues by combining cells with porous scaffolds materials. Scaffolds are templates for tissue regeneration and should ensure suitable cell adhesion and mechanical stability throughout the application period. Chitosan (CS) is a biocompatible polymer highly investigated for scaffold preparation but suffers from poor mechanical strength. In this study, graphene oxide (GO) was conjugated to chitosan at two weight ratios 0.3% and 1%, and the resulting conjugates were used to prepare composite scaffolds with improved mechanical strength. To study the effect of GO oxidation degree on scaffold mechanical and biological properties, GO samples at two different oxygen contents were employed. The obtained GO/CS scaffolds were highly porous and showed good swelling in water, though to a lesser extent than pure CS scaffold. In contrast, GO increased scaffold thermal stability and mechanical strength with respect to pure CS, especially when the GO at low oxygen content was used. The scaffold in vitro cytocompatibility using human primary dermal fibroblasts was also affected by the type of used GO. Specifically, the GO with less content of oxygen provided the scaffold with the best biocompatibility.

## 1. Introduction

Chitosan is a cationic polysaccharide, deriving from chitin deacetylation, which has gained a prominent place in biomedicine for a wide range of applications including drug delivery, wound dressings, bacterial contamination control, fat binding, and tissue engineering [1,2,3,4,5]. The peculiarity of chitosan, compared to other polysaccharides, is that it has been shown to provoke minimal or no foreign-body reaction, including inflammatory response and fibrotic encapsulation when used in hydrogel systems [6,7], polyelectrolyte multilayers [8], biomembranes [9] and as a porous 3-D scaffold [10]. Besides, chitosan has been shown to promote cell adhesion and proliferation in tissue engineering applications, especially when applied for bone tissue regeneration where it showed osteoconductivity and ability to promote osteogenic differentiation [11,12,13,14,15,16].

The major limitation of chitosan for the repair of bone defects is its low mechanical strength, which precludes pure chitosan scaffolds for load-bearing applications. For such reason, many chitosan composite scaffolds have been lately developed to improve mechanical scaffold properties and bioactivity [16,17]. Main substances used in combination with chitosan to produce scaffolds for bone tissue regeneration are tricalcium phosphate [18], hydroxyapatite [19], silica nanoparticles [20], and, more recently, graphene oxide [21,22,23,24,25]. Particularly, graphene oxide (GO) is obtained by oxidation and exfoliation of graphite [26,27] and consists of a monolayer of sp^2^-hybridized carbon atoms arranged in a honeycomb structure decorated with oxygen-containing groups, including hydroxyl, epoxy and carboxylic groups [28]. Such material is considered highly promising for bone tissue engineering because not only it presents high mechanical stiffness and flexibility, but it was also shown to improve osteogenesis and cellular differentiation when combined with other biomaterials [23,29,30,31,32]. GO also showed significant antibacterial activity [33] like other 2D-nanomaterials [34]. Therefore, its incorporation into the chitosan matrix opens perspectives for antimicrobial applications of GO-chitosan scaffolds.

A series of different types of GO can be produced, which may vary in terms of layer surface area, structural defects, sp^2^/sp^3^ ratio and oxygen content. Specifically, the oxygen content, which strongly depends on the GO preparation method and post-oxidation treatments [35,36], can significantly affect GO mechanical properties, conductivity, ability to disperse in water and biocompatibility. Indeed, oxygen groups present on the basal plane and edges of GO enable it to interact with cellular components like proteins, mainly through electrostatic interaction and hydrogen bonds [37,38,39].

In this study, in order to investigate the effects of GO oxygen content on scaffold mechanical and biological properties, 3D porous scaffolds based on chitosan/graphene oxide conjugates were prepared by employing two types of GO, a GO sample at low oxygen content (commercially available) and a GO sample at high oxygen content obtained by electrochemical exfoliation of graphite. GO was conjugated to chitosan by a carbodiimide-mediated amidation in two concentrations (0.3% and 1%). Then, scaffolds, prepared by either the salt leaching method or freeze-drying, were characterized in terms of water swelling, water retention ability, thermal properties, mechanical response in compression and in vitro cytocompatibility against primary human dermal fibroblasts.

## 2. Results and Discussion

### 2.1. GO Characterization

**Figure 1:**
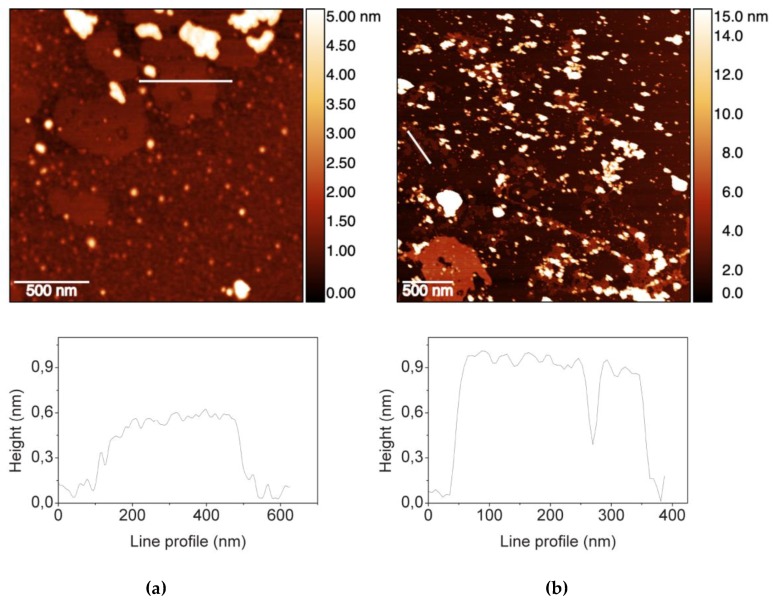
Atomic Force Microscopy (AFM) topographies of: (**a**) GO_LiClO_4_ sample (GO_exfoliated_); (**b**) commercial GO product (GO_sigma_) and related height profiles.

Figure 1a shows the Atomic Force Microscopy (AFM) topography of GO_LiClO_4_, named GO_exfoliated_. This sample exhibits small surface area (ranging from 0.03 to 0.15 µm^2^) and the average height in the range of 3–4 ML (Mono Layer), although also single layer sheets (ca. 0.6 nm in thickness, as shown in Figure 1a) are often observed. Indeed, a single layer of GO on mica typically exhibits a 0.6–1.0 nm height, greater than the graphene thickness. 

Figure 1b exhibits the AFM topography of commercial GO product, named GO_sigma_. The latter is characterized by a graphene sheet with well homogeneous in thickness but heterogeneous in area distribution (0.006–0.010 µm^2^). In 90% of cases, the sheets are 1–2 layer thick (Figure 1b). Thicker sheets are less frequent but generally wider. 

**Figure 2:**
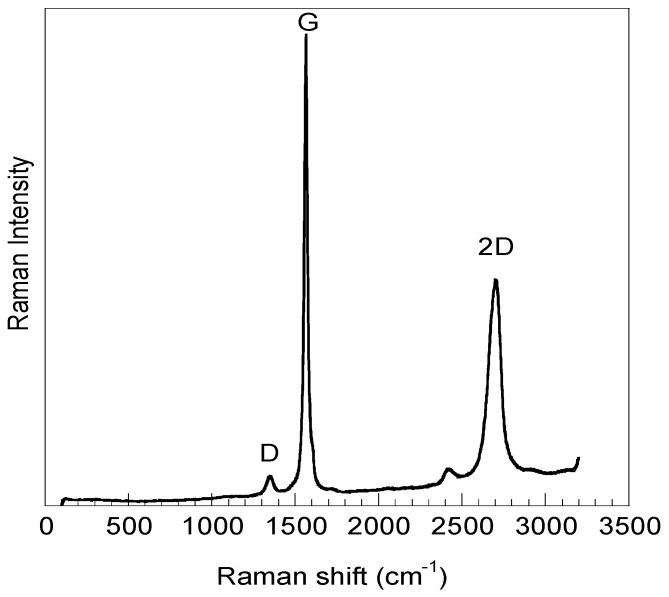
Raman spectrum of GO_sigma_.

Figure 2 shows the Raman spectrum of GO_sigma_, while Table 1 reports the Raman parameters for both samples. The Raman spectrum of GO_exfoliated_ was already published [40].

In the spectrum, the typical fingerprint of graphene is observable, with G-band at around 1580 cm^−1^, corresponding to the first-order scattering of the E2g vibration mode, and a 2D band at 2700 cm^−1^, corresponding to the second-order two phonon mode. Moreover, a D-band is present at 1350 cm^−1^, reflecting the presence of structural defects (vacancies, edge defects, heteroatoms, etc.) [41]. The intensity ratio of D and G bands (I_D_/I_G_), reported in Table 1, provides information about structural defects on graphene surface and edges. Between the two samples, GO_sigma_ has the lowest I_D_/I_G_ ratio indicating that this sample has the lowest content of defects.

**Figure 3:**
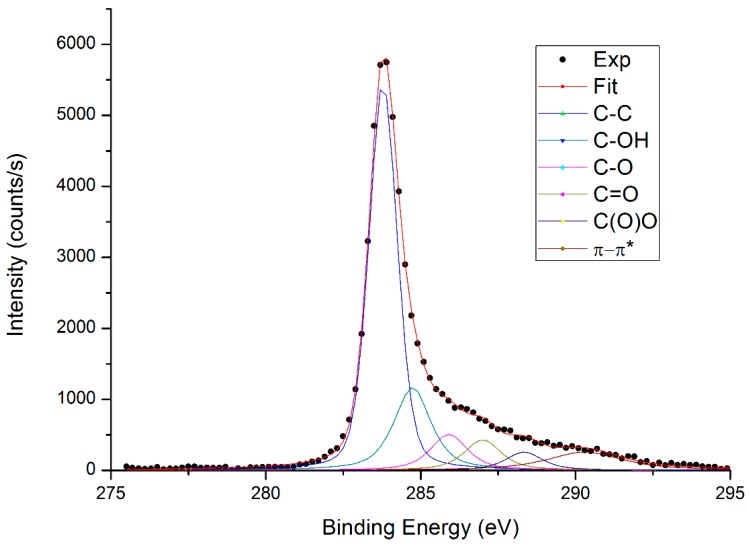
X-ray Photoelectron Spectroscopy (XPS) C1s spectrum of GO_sigma_.

In agreement with such data, XPS analysis revealed a lower content of oxygenated groups in the GO_sigma_ than the electrochemical synthesized GO_exfoliated_ (Table 2). In Figure 3, the XPS spectrum of commercial GO is reported, while the XPS spectrum of GO_exfoliated_ has already been published [40].

In Table 2, the deconvolution results of the relative C1s peaks are reported for both samples. The presence of aromatic C=C and aliphatic C–C is demonstrated by the main peak at binding energy 283.8 eV and the π–π* peak at ca. 291 eV attributed π-electrons delocalized in the aromatic network. The C–C peak was fitted with a weighted Voigtian profile instead of a Doniach-Sunjic one to take into account the contribution of C–H group too close to the C–C to be deconvolved singularly. All samples also showed a significant oxidation degree (i.e. functionalization degree), as indicated by the presence of hydroxyl, epoxide, carbonyl, and carboxyl functional groups. Between the two samples, the non-oxidized carbon component was more intense for GO_sigma_; while GO_exfoliated_ exhibited the highest content of oxygen-containing functional groups, and therefore the highest degree of oxidation/functionalization. This difference in oxidation degree could affect the physico-chemical properties of the resulting chitosan/GO composite materials and, thus their application.

### 2.2. Preparation and Characterization of GO/CS Composite Scaffolds

**Figure 4:**
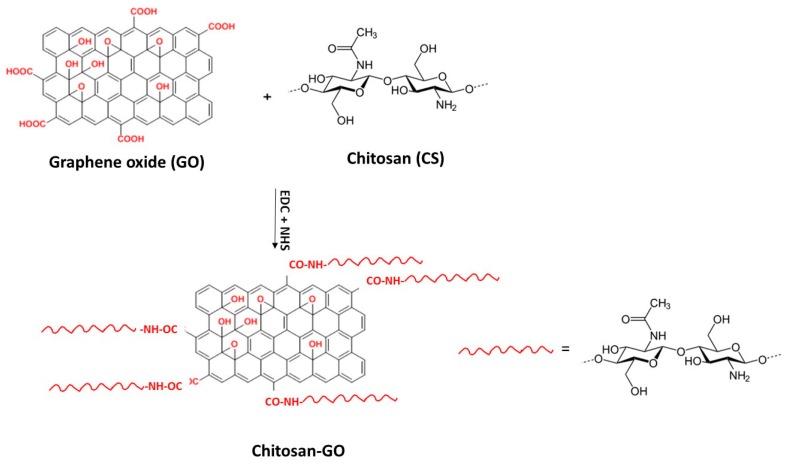
Scheme of Chitosan-GO amidation reaction.

In this study, the two characterized GO samples were used in combination with chitosan to prepare composite scaffolds for application in tissue engineering. To achieve intimate contact between the polymeric matrix and the filler, GO was covalently linked to chitosan by an amidation reaction (Figure 4).

Two GO/CS weight ratios (0.3% and 1%) were used during CS functionalization reaction. These concentrations were chosen as a compromise to obtain scaffolds combining good mechanical and biological properties. Indeed, in the literature, it was found that GO contents higher than 1% in chitosan based-scaffolds could only slightly improve mechanical properties [42] or even worsen them in terms of compressive strength [24]. Also, cell viability could be altered for too high GO content [42].

**Figure 5:**
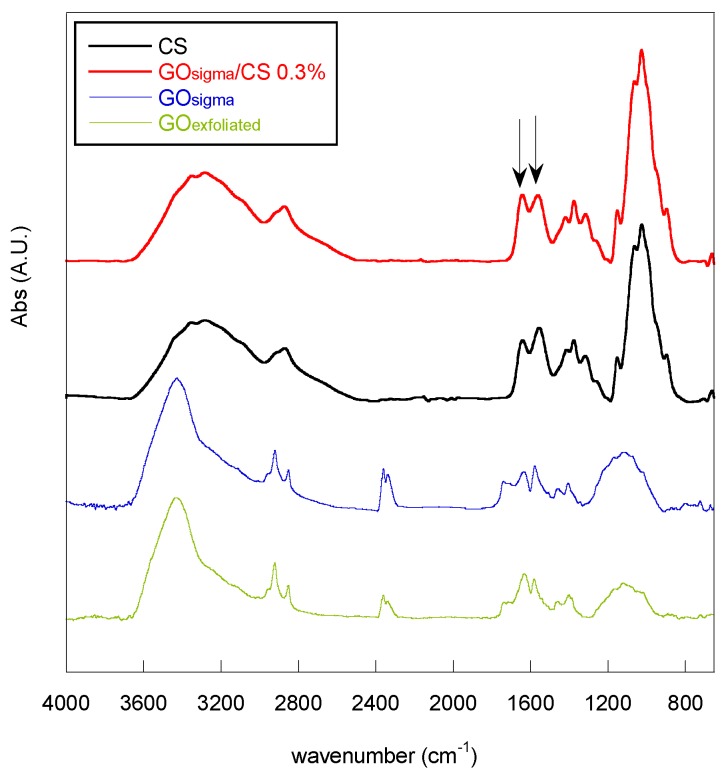
FTIR spectra of GO_sigma_, GO_exfoliated_, chitosan and of the GO-functionalized chitosan obtained with GO_sigma_, and a GO/CS weight ratio of 0.3%.

FTIR IR-ATR analysis confirmed qualitatively the success of amidation reaction in all of the used experimental conditions and for both types of GO. In Figure 5, as an example, the IR-ATR spectra of CS and of the GO-functionalized chitosan obtained with the GO_sigma_—at a 0.3% GO/CS weight ratio—are reported. The spectra of GO_sigma_ and GO_exfoliated_ were also reported for comparison. In both spectra, the absorption peaks of oxygenated groups are present (3427 cm^−1^ stretching O–H; 1738 stretching C=O; 1050–1280 cm^−1^ stretching C–O–C). As for the amidation reaction, the peaks at 1644 cm^−1^ and at 1560 cm^−1^, attributed to stretching of C=O amide of CS and bending N–H of CS primary amine respectively, are present in the spectrum of the GO-functionalized chitosan but in a different ratio with respect to pure chitosan. Specifically, in all of the chitosan derivatives, an increase in the intensity ratio A_1644_/A_1560_ was observed (Table 3), which is presumably related to a decrease in the number of NH_2_ (decrease in the intensity of the peak at 1560 cm^−1^) as a consequence of amidation with GO. However, physical interactions between chitosan and GO cannot be excluded, and they can occur to some extent [43].

The GO-functionalized chitosan derivatives were then used to prepare porous scaffolds by the freeze-drying method or the salt leaching method. In Table 4, the types of prepared scaffolds and corresponding acronyms were reported.

**Figure 6:**
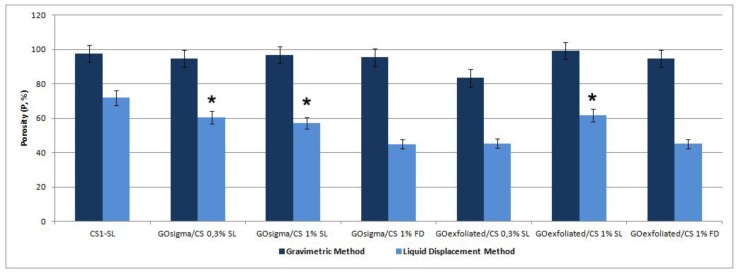
Porosity (%) of all the prepared scaffolds. The symbol (*) indicates samples with the best interconnected porosity.

In Figure 6, the porosity determined with either the gravimetric method or the liquid displacement method for all the prepared scaffolds is reported.

As it can be observed, the porosity values determined by the gravimetric method were quite high (an average of 95% for all of the systems) and always greater than those determined by the liquid displacement method. That is because the gravimetric method provides information about the total scaffold porosity while the second method only about the pore fraction accessible to the liquid (ethanol), that is the fraction of interconnected pores. Some differences were observed in terms of such fraction among the various scaffolds.

Specifically, for both types of GO, the salt leaching permitted to obtain scaffold with a greater fraction of interconnected pores compared to freeze-drying. The GO content (0.3% or 1%) only slightly affected pore interconnection in the case of GO_exfoliated_.

**Figure 7:**
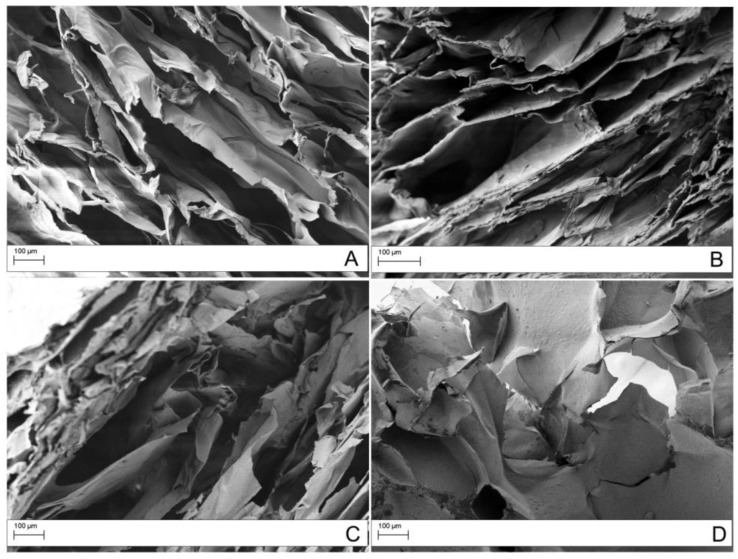
Field Emission Scanning Electron Microscope (FESEM) micrographs of scaffolds obtained with 1% CS solution: (**A**) CS; (**B**) GO_sigma_/CS_SL_ 1%; (**C**) GO_exfoliated_/CS_SL_ 1%; (**D**) GO_sigma_/CS_FD_ 1%.

Field Emission Scanning Electron Microscope (FESEM) observations confirmed the higher level of porosity of the scaffolds prepared with the salt leaching method compared to the freeze-drying (Figure 7). 

**Figure 8:**
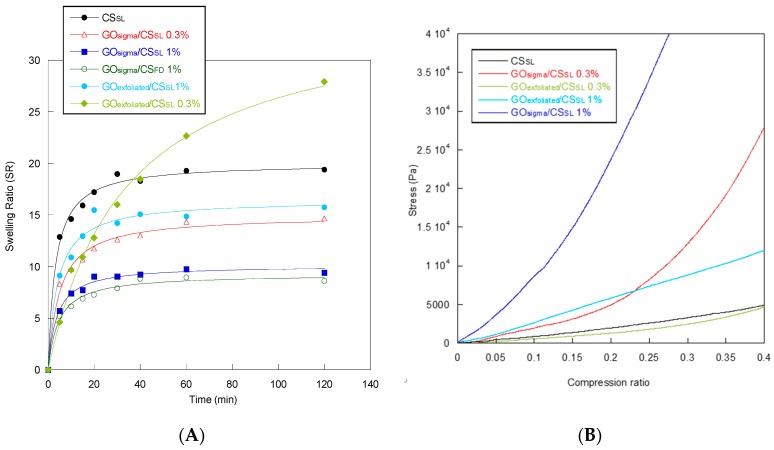
Swelling ratio (**A**) and mechanical behavior in compression tests (**B**) of the scaffolds.

Water swelling and water retention efficiencies are important properties for a scaffold since a low swelling degree may hamper nutrient diffusion through the scaffold while a high swelling degree may compromise scaffold integrity over usage [44]. As shown in Figure 8A, all of the scaffolds absorbed water very quickly, reaching the equilibrium in approximately 20 minutes. Composite scaffolds showed a lower swelling degree than pure CS scaffold, with the only exception of GO_exfoliated_/CS_SL_ 0.3%. For a fixed GO concentration (0.3% or 1%), the GO_exfoliated_ make the scaffolds more hydrophilic than the GO_sigma_, due to its high content of oxygenated groups. For a fixed type of GO (GO_exfoliated_ or GO_sigma_), instead, an increase in GO concentration decreased scaffold hydrophilicity because of the hydrophobicity of the aromatic C=C of the graphene plane. As reported in Table 5, the highest equilibrium swelling ratio was shown by the GO_exfoliated_/CS_SL_ 0.3% sample. A reduced degree of swelling of the composite scaffolds compared to CS could contribute to improving scaffold mechanical stability while the high initial rate of swelling could ensure a suitable supply of nutrients to cells seeded in the scaffold [45].

As far as the water retention efficiency is concerned, the composite GO/CS scaffolds had sufficiently high water retention (WR) values ranging from 4 of the scaffolds obtained by freeze drying to 8 of GO_exfoliated_/CS_SL_ 0.3%, this latter being the sample also showing the highest swelling in water (Table 5). WR decreased with increasing GO content presumably because a higher CS functionalization resulted in a decreased number of CS amino groups available for interaction with water. In agreement with such a hypothesis, the scaffold made of pure CS showed the highest WR value (Table 5).

Thermal properties of the GO/CS composite scaffolds were studied by thermogravimetric analysis and differential scanning calorimetry. In Table 5, the degradation temperature (T_d_) and glass transition temperature (T_g_) of chitosan and GO/CS composite scaffolds are reported. As it can be observed, the composite scaffolds showed T_d_ values higher than that of CS, suggesting the formation of a crosslinked structure where presumably GO forms bridges among the polymer chains. Such network seemed to be stronger with increasing the GO content. The values of the glass transition temperature of the GO/CS composite scaffolds (Table 5) are also coherent with the formation of a cross-linked structure induced by CS amidation with GO. Indeed, all of the composite scaffolds showed a T_g_ higher than CS indicating a reduced polymer chain mobility.

Finally, the mechanical behavior in compression of the scaffolds was studied. In Figure 8B the stress versus the compression ratio is reported for CS and composite scaffolds while in Table 5 the Compressive Modulus for all of the scaffolds is reported.

The composite scaffolds showed Compressive Modulus values greater than CS_SL_ scaffold, especially at 1% GO content. The only exception was GO_exfoliated_/CS_SL_ 0.3% that had a Compressive Modulus similar to CS_SL_. At a fixed GO content, either 0.3% or 1%, the scaffolds obtained with the GO_sigma_ were stiffer than those obtained with GO_exfoliated_, suggesting a better dispersion of GO_sigma_ in the CS structure. Presumably, the average size of GO_sigma_ sheets smaller than GO_exfoliated_, as resulted from AFM observations, contributed to the observed better dispersion and enhanced mechanical properties of the GO_sigma_/CS scaffolds [46,47]. Aggregation phenomenon in GO_exfoliated_ could also be related to the formation of hydrogen bonds among oxygenated functional groups present in the sheet basal planes, mediated by water molecules entrapped within the interlayer cavities. Indeed, it is known that a hydrogen bond network is present among GO sheets and water molecules even after prolonged drying [48,49,50]. The scaffold’s mechanical properties could also be affected by the extent of the amidation reaction. In our case, even if the amidation degree was not quantified, it can be hypothesized that GO_exfoliated_ reacted more efficiently with CS leading to a higher GO incorporation for a fixed GO/CS weight ratio. That could contribute to increasing GO aggregation phenomena in GO_exfoliated_/CS scaffolds with a consequent decrease in the scaffold’s mechanical properties, as found by Sivashankari et al. in scaffolds of hydroxypropyl chitosan-graft-graphene oxide [24].

### 2.3. Assessment of Cell Viability in the Scaffolds 

**Figure 9:**
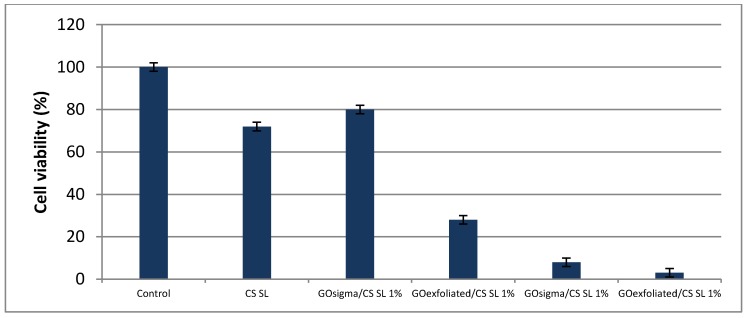
Cell viability on CS and composite scaffolds.

The biocompatibility of the scaffolds was evaluated on human dermal fibroblasts by a mitochondrial activity-based assay that uses the tetrazolium dye [3-(4,5-dimethylthiazol-2-yl)-5-(3-carboxymethoxyphenyl)-2-(4-sulfophenyl)-2H-tetrazolium] MTS. Figure 9 exhibits the viability of the cells in the presence of scaffolds after 48 h of culture. As can be seen, pure chitosan showed good biocompatibility with cell viability of ca 75% compared to control. In contrast, pristine GO_exfoliated_ and GO_sigma_ samples were found to be toxic for the cells, especially GO_exfoliated_ having the highest oxygen content.

Literature data on GO cytotoxicity are controversial since several factors can affect the cytocompatibility of such material including concentration, size, shape and oxygen content [51]. A greater hemolytic activity was shown by GO at high oxygen content compared to graphene sheets [51]. Similarly, GO was shown to generate more reactive oxygen species (ROS) than reduced GO materials (lower oxygen content) when tested against murine lung epithelial cells [52]. However, the toxicity of GO can be drastically reduced by coating it with biocompatible polymers like polyvinylpyrrolidone [53], collagen [54] and chitosan [25,51].

Also in our case, biocompatibility of composite scaffolds was affected by the type of used GO. Specifically, the GO at low oxygen content (GO_sigma_) increased biocompatibility of CS scaffold; cell viability being ca. 80% compared to 75% of pure CS (Figure 9). That was not true for GO_exfoliated_, for which a reduction of scaffold biocompatibility was observed following GO incorporation. Presumably, in the case of GO_exfoliated_, GO aggregates present within the composite structure were not adequately shielded by CS to avoid interaction with cells. That would be in agreement with the observed lower mechanical strength of the GO_exfoliated_/CS scaffolds compared with the GO_sigma_/CS series.

**Figure 10:**
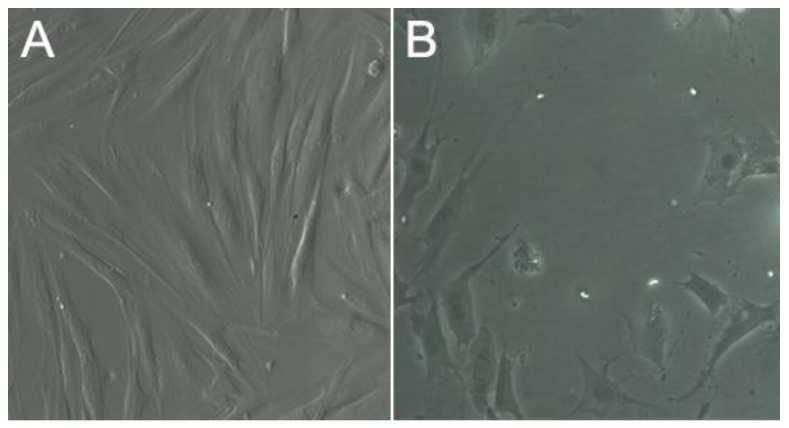
Optical images of fibroblast cells grown in the absence (**A**) or in the presence (**B**) of the scaffold GO_sigma_/CS_SL_ 1%.

In order to study possible cell morphology changes induced by contact with GO/CS scaffold, in Figure 10, as an example, the optical images of cells cultured on 96 well plates compared to those remaining in the plate after contact with the scaffold GO_sigma_/CS_SL_ 1% are reported. 

As it can be observed, cells grown in contact with the scaffold did not present signs of damage (Figure 10B). Only a slight morphology change was observed with a less elongated and more enlarged cell shape (Figure 10B) with respect to control (Figure 10A). That finding is doubtless related to the low number of cells remained in the culture plate due to penetration of seeded cells in the scaffold. Indeed, such a low number of cells could distribute in a larger area compared to control.

## 3. Materials and Methods

### 3.1. Graphene Oxide 

Two types of graphene oxide (GO) were used in this study: a) Graphene oxide from Sigma-Aldrich (Merck KGaA, Darmstadt, Germany; 4–10% edge-oxidized, with 15–20 number of layers, 1.8 g/cm^3^ bulk density), named GO_sigma_; b) Graphene oxide prepared by electrochemical exfoliation of graphite using the salt KClO_4_ in the electrolytic solution, as previously described [40]. This sample was named GO_exfoliated_.

### 3.2. Characterization of Graphene Oxide Samples

The morphology of the GO sheets was evaluated by Atomic Force Microscopy (AFM) using a Veeco AFM Multimode™ (Veeco, Plainview, NY, USA) equipped with a Nanoscope IIIa controller. For the analysis, a drop (10 µl) of a GO dispersed in deionized water (0.01 mg·mL^−1^) was layered onto a clean silicon wafer with negligible roughness. All images were obtained in tapping mode acquiring topography, amplitude and phase data, by using a Rectangular Tip Etched Silicon Probe (RTESP, Bruker, Billerica, MA, USA; nominal parameters r = 8 mm, f = 300 kHz, k = 40 N/m) and with a 512 × 512 pixels resolution. The software Gwyddion 2.31 (Version 2.31, Gwyddion, Brno, Czech Republic, http://gwyddion.net/) was used to correct images by polynomial background filters and to calculate average thickness and dimensions of GO sheets.

The presence of functional groups on GO edges and basal planes was investigated by Fourier transform infrared spectroscopy (FTIR) and X-ray Photoelectron Spectroscopy (XPS). FTIR spectra were acquired in transmission by a Nicolet 6700 FTIR (Thermo Fisher Scientific, Waltham, MA, USA), by co-adding 100 scans at a resolution of 2 cm^−1^. GO powder (ca. 1 mg) was pelleted in 150 mg of KBr using a Specac manual hydraulic press, by applying a pressure of 2 tons for 5 min. XPS was performed by an Omicron DAR 400 Al/Mg Kα non-monochromatized X-ray source (Scienta Omicron GmbH, Taunusstein, Germany), and a VG-CLAM2 electron spectrometer (Thermo Fisher Scientific, Waltham, MA, USA). For the analysis, GO was dispersed in ethanol to a 1 mg·mL^−1^ content and deposited onto a silicon wafer.

The carbon structure of GO sheets was analyzed by Raman Spectroscopy. Raman spectrometer XY Dilor (HORIBA Jobin Yvon GmbH, Bensheim, Germany), recording the spectrum from 1200 to 2900 cm^−1^, with a resolution of 2 cm^−1^, using an excitation wavelength of 514.5 nm. The power of the laser beam was 3.5 mW, focused on the sample by using a 100× objective and performing 10 repetitions of 60 s, for each measurement. 

### 3.3. Functionalization of Chitosan with GO

GO was covalently linked to chitosan (CS, Sigma Aldrich, Merck KGaA, Darmstadt, Germany; medium molecular weight, 75–85% deacetylated) by amidation between GO carboxylic groups and CS amino groups (Figure 4). First, chitosan was dissolved in 1% acetic acid aqueous solution at 1% (w/v) concentration. CS solution was then dialyzed in water (membrane cutoff = 3.5 KDa) to remove acetic acid and low molecular weight by-products.

The determined amount of GO was suspended in water and exfoliated by sonication for 4 h at 40°C. Then, GO carboxylic groups were activated by 1-Ethyl-3-(3-dimethylaminopropyl) carbodiimide (EDC, Sigma Aldrich, Merck KGaA, Darmstadt, Germany) and N-Hydroxysuccinimide (NHS, Sigma Aldrich, Merck KGaA, Darmstadt, Germany), added in amounts such to achieve a 0.1 M concentration of each. After 2 h of activation at room temperature, chitosan solution at 1% (w/v) concentration was added to the GO suspension such to have a GO/CS weight ratio of either 0.3% or 1%. The amidation was carried out under stirring at room temperature for 24 h.

Following reaction, CS/GO suspensions were centrifuged at 3500 RPM for 10 min to eliminate the unreacted GO; then the supernatant was recovered and dried under vacuum. The obtained polymer samples were named GO_X_/CS Y% where X was the type of employed GO (sigma or exfoliated), and Y was the GO/CS weight ratio used for CS amidation (0.3% or 1%).

The amidation reaction was followed by FTIR analysis. Spectra were acquired in attenuated total reflection (ATR) by a Nicolet 6700 (Thermo Fisher Scientific, Waltham, MA, USA) equipped with a Golden Gate single reflection diamond ATR accessory at a resolution of 2 cm^−1^ and co-adding 100 scans. 

### 3.4. Preparation of GO/CS Composite Scaffolds

Porous GO/CS composite scaffolds were prepared by employing two methods, the freeze-drying (FD) and the salt leaching (SL). In the first method, after the reaction, the solution of GO-functionalized chitosan was poured into a steel mold with a square base and frozen in liquid nitrogen. Then, the frozen polymer was removed from the mold and lyophilized for 1 day. In the second method, a porogen, sodium acetate (100–200 µm), was added in the solution of GO-functionalized chitosan. After stirring for 1 h, the solution of the GO-functionalized chitosan containing the porogen was poured into a steel mold, frozen and lyophilized. Then, the lyophilized polymer was immersed first in ethanol/water solutions (96%, 80%, 60%, 40% v/v), 2 h for each concentration, and then in water for 48 h to remove the salt and form a porous structure. Finally, the wet porous scaffold was dried by lyophilization. A scaffold of pure chitosan was also prepared by the salt leaching method employing a 1% CS solution. 

The obtained scaffolds were named as follow: GO_X_/CS_M_ Y% where X was the type of employed GO (sigma or exfoliated), M was the method used to prepare the scaffold (FD or SL), and Y was the GO/CS weight ratio used for CS amidation (0.3% or 1%). In Table 4, all the prepared samples with the corresponding acronyms are reported.

### 3.5. Characterization of GO/CS Composite Scaffolds

The porosity of GO/CS composite scaffolds was measured by the gravimetric method [55] and the liquid displacement method [56]. 

The gravimetric method permits to evaluate the scaffold porosity (*P*) by determining the bulk and true density of the scaffold as shown in the equations below:

(1) $$\rho_{s}=\frac{m}{V}$$

(2) $$P\left(\%\right)=\left(1-\frac{\rho_{s}}{\rho_{c}}\right)\cdot 100$$

where $\rho_{s}$ is the apparent density of the scaffold, m is the weight of the scaffold, V is the volume of the scaffold, and $\rho_{m}$ is the density of the material used to prepare the scaffold, in our case chitosan. Chitosan was considered to have a density of 1.41 g/cm^3^.

As for the liquid displacement method, ethanol was used as the displacement liquid because it penetrated easily into the pores and, being a non-solvent of chitosan, did not induce shrinkage or swelling of the scaffold. A scaffold sample with the initial weight *W*_0_ and initial volume *V*_0_ was immersed for 30 min in a cylinder containing a known volume of ethanol (*V*_1_). Then, the scaffold was removed and weighed (*W*_1_). Sample porosity (*P*) was calculated as follow:

(3) $$P\left(\%\right)=\left(\frac{W_{1}-W_{0}}{\rho_{EtOH}\cdot V_{0}}\right)\cdot 100$$

where $\rho_{EtOH}$ is the density of ethanol (0.806 g/cm^3^ a 20 °C).

The microstructure of the scaffolds were observed by field emission scanning electron microscope (FESEM, AURIGA Carl Zeiss AG, Oberkochen, Germany). For the analysis, the scaffolds were fractured in liquid nitrogen, then fixed on stubs and gold sputter before observation.

Water uptake of scaffolds was determined at room temperature by immersing the scaffolds in water for increasing times. At determined intervals, scaffolds were removed from water and weighed, after removal of the excess of solvent using filter paper. The analysis was repeated until constant weight (equilibrium swelling weight, *W*′) was reached. The swelling ratio, *SR*, was calculated by applying the following equation:

(4) $$SR=\left(\frac{W_{t}-W_{0}}{W_{0}}\right)$$

where *W_t_* was the weight of the sample after swelling at the time t and *W*_0_ was the initial weight of the film. Five parallel swelling experiments were performed for each sample and data were reported as average value ± standard deviation.

The water retention efficiency was determined by transferring the swollen scaffold, with the maximum swelling (*W*′), into a centrifuge tube having a filter paper at the bottom. Then the sample was centrifuged at 500 rpm for 3 min and immediately weighed (*W_f_*). Water retention (*WR*) of the scaffold was calculated as follows:

(5) $$WR=\frac{W^{\prime }-W_{f}}{W^{\prime }}$$

Differential scanning calorimetry (DSC) was performed from −100 to +150 °C under N_2_ flux by using a Mettler TA-3000 DSC apparatus (Mettler Toledo, Columbus, OH, USA). The scan rate used for the experiments was 10 °C·min^−1^ and the sample weight of 6–7 mg. Thermo-gravimetric analysis (TGA) was carried out employing a Mettler TG 50 thermobalance (Mettler Toledo, Columbus, Ohio, Stati Uniti) at a heating rate of 10°C·min^−1^ under N_2_ flow in the temperature range 25–600°C.

The compressive strength of scaffolds was determined by an ISTRON 4502 instrument (INSTRON, Norwood, MA, USA). Measurements were performed on parallelepipeds 10 mm height with a square base (5 mm × 5 mm), obtained by using a proper steel mold. Particularly, solutions of GO-functionalized chitosan were poured into the mold and frozen in liquid nitrogen. Then, the frozen samples were removed from the mold and lyophilized for 1 day. When a porogen was used, the lyophilized samples were immersed in water/ethanol solutions to remove the porogen (see Section 3.4) and lyophilized again. The crosshead speed of the Instron tester was set at 1 mm/min, and load was applied until 40% reduction in specimen height. Five parallel samples were tested for every scaffold, and mechanical properties were reported as average value ± standard deviation. 

### 3.6. Assessment of Cell Viability in Scaffolds by MTS Assay

Cell compatibility and cytotoxicity were analyzed by culturing human primary dermal fibroblasts in the presence of the scaffolds. Cells were obtained from young adult male patients complaining of phimosis, full ethical consent was obtained from all donors and the Research Ethics Committee, Sapienza University of Roma, approved the study. Scaffolds were set down in 96 well tissue culture plate and conditioned in Dulbecco’s Modified Eagle’s Medium DMEM without red phenol supplemented with L-glutamine, penicillin/streptomycin, Na-pyruvate, non-essential amino acids, plus 10% Fetal Bovine Serum (FBS) for 2 hours at 37°C, in 95% humidity and 5% CO_2_ atmosphere. Equal numbers (8 × 10^3^) of cells were seeded in each well containing the scaffolds and allowed to proliferate for 48h. Cellular viability/proliferation was quantified by measuring the mitochondrial dehydrogenase activity using tetrazolium dye MTS [3-(4,5-dimethylthiazol-2-yl)-5-(3-carboxymethoxyphenyl)-2-(4-sulfophenyl)-2H-tetrazolium] (Promega Corporation, Madison, WI, USA) based colorimetric assay, according to the manufacturer’s instructions. Briefly, after 48 h, 20% (v/v) of MTS dye was added in the culture media and cells were cultured for 4 h to allow the formation of soluble formazan crystals by viable cells. Spectrophotometric absorbance was measured at 490 nm using a multi-plate reader Appliskan (Thermo Fisher, Waltham, MA, USA). Cells cultured in the absence of the scaffolds were taken as a control. In order to analyze the cytotoxicity of pristine GO, human primary fibroblasts (8 × 10^3^) were cultured in the presence of 0.1 μg/μl (final concentration) powder of both GO_exfoliated_ and GO_sigma_ and then the cells were analyzed as described above with MTS dye.

## 4. Conclusions

This study confirms the ability of graphene oxide to act as reinforcing filler for chitosan scaffolds. Findings suggest that the oxygen content of GO affects the final properties of GO/CS composite scaffolds. Specifically, high oxygen content in GO can promote aggregation of GO sheets in the chitosan matrix and, hence, reduce its reinforcement effect. Additionally, high content of functional groups in GO have a negative effect on material biocompatibility, presumably because they could promote the generation of reactive oxygen species as reported in the literature. In contrast, the conjugation of chitosan with a GO sample at low oxygen content resulted in scaffolds with improved compression modulus and biocompatibility compared to pristine CS. Overall, GO_sigma_/CS scaffold at 1% GO content showed good potentiality for application in tissue engineering.

## Figures and Tables

**Table 1:** Raman parameters for GO_sigma_ and GO_exfoliated_.

Sample	Frequency (cm^−1^)	Assigned Bands	I_D_/I_G_
GO_sigma_	2706	2D	0.06
	1565	G	
	1350	D	
GO_exfoliated_	2713	2D	0.27
	1582	G	
	1357	D	

**Table 2:** Atomic (At) percentages from C1s fit for GO_sigma_ and GO_exfoliated_.

Peak BE (eV)	Species	GO_sigma_ At (%)	GO_exfoliated_ At (%)
283.8	C–C	60.7	50.0
284.7	C–OH	21.7	18.0
285.9	C–O	9.4	16.0
287.0	C=O	8.2	9.0
289.0	C(=O)O	4.3	7.0

**Table 3:** Intensity ratio of peaks at 1644 cm^−1^ and 1560 cm^−1^ (A_1644_/A_1560_) for the GO/CS samples.

Sample	CS Conc. (%, w/v)	A_1644_/A_1560_
CS	–	0.82
GO_sigma_/CS 0.3%	1	0.93
GO_sigma_/CS 1%	1	1.02
GO_exfoliated_/CS 0.3%	1	0.88
GO_exfoliated_/CS 1%	1	0.91

**Table 4:** Types of prepared scaffolds and corresponding acronyms.

Acronym	Method for Scaffold Preparation	GO/CS % (w/w)
CS_SL_	SL *	–
GO_sigma_/CS_SL_ 0.3%	SL	0.3
GO_sigma_/CS_SL_ 1%	SL	1
GO_sigma_/CS_FD_ 1%	FD *	1
GO_exfoliated_/CS_SL_ 0.3%	SL	0.3
GO_exfoliated_/CS_SL_ 1%	SL	1
GO_exfoliated_/CS_FD_ 1%	FD	1

* SL = salt leaching method; FD = Freeze-drying method.

**Table 5:** Equilibrium swelling ratio (SW), water retention (WR), degradation temperature (T_d_) and glass transition temperature (T_g_) of chitosan and GO/CS composite scaffolds.

Sample	Equilibrium Swelling Ratio (SR)	Water Retention (WR)	T_d_ (°C)	T_g_ (°C)	Compressive Modulus (KPa)
CS_SL_	19 ± 2	10.1 ± 0.5	280	72	12 ± 3
GO_sigma_/CS_SL_ 0.3%	14 ± 1	7.1 ± 0.5	296	84	22 ± 2
GO_sigma_/CS_SL_ 1%	9 ± 2	5.9 ± 0.5	304	82	108 ± 5
GO_sigma_/CS_FD_ 1%	8 ± 1	4.2 ± 0.5	ND *	ND	ND
GO_exfoliated_/CS_SL_ 0.3%	28 ± 4	7.6 ± 0.5	283	86	10 ± 3
GO_exfoliated_/CS_SL_ 1%	16 ± 3	5.5 ± 0.5	286	84	31 ± 4
GO_exfoliated_/CS_FD_ 1%	10 ± 2	4.0 ± 0.5	ND	ND	ND

* ND = Not determined.
